# Impact of Perception Errors in Vision-Based Detection and Tracking Pipelines on Pedestrian Trajectory Prediction in Autonomous Driving Systems

**DOI:** 10.3390/s24155066

**Published:** 2024-08-05

**Authors:** Wen-Hui Chen, Jiann-Cherng Wu, Yury Davydov, Wei-Chen Yeh, Yu-Chen Lin

**Affiliations:** 1 Graduate Institute of Automation Technology, National Taipei University of Technology, Taipei 10608, Taiwan; whchen@ntut.edu.tw (W.-H.C.);; 2 Department of Automatic Control Engineering, Feng Chia University, Taichung 40724, Taiwan

**Keywords:** autonomous driving, trajectory prediction, object detection, object tracking

## Abstract

Pedestrian trajectory prediction is crucial for developing collision avoidance algorithms in autonomous driving systems, aiming to predict the future movement of the detected pedestrians based on their past trajectories. The traditional methods for pedestrian trajectory prediction involve a sequence of tasks, including detection and tracking to gather the historical movement of the observed pedestrians. Consequently, the accuracy of trajectory prediction heavily relies on the accuracy of the detection and tracking models, making it susceptible to their performance. The prior research in trajectory prediction has mainly assessed the model performance using public datasets, which often overlook the errors originating from detection and tracking models. This oversight fails to capture the real-world scenario of inevitable detection and tracking inaccuracies. In this study, we investigate the cumulative effect of errors within integrated detection, tracking, and trajectory prediction pipelines. Through empirical analysis, we examine the errors introduced at each stage of the pipeline and assess their collective impact on the trajectory prediction accuracy. We evaluate these models across various custom datasets collected in Taiwan to provide a comprehensive assessment. Our analysis of the results derived from these integrated pipelines illuminates the significant influence of detection and tracking errors on downstream tasks, such as trajectory prediction and distance estimation.

## 1. Introduction

In the area of autonomous systems [1,2,3], accurate pedestrian trajectory prediction and reliable distance estimation are two crucial components that play important roles in ensuring safe and efficient navigation. Trajectory prediction has many potential applications and can also be utilized in robotics [4]. Pedestrian trajectory prediction allows autonomous vehicles to anticipate the future movements of pedestrians, enabling timely and proactive responses to potential collision scenarios. On the other hand, distance estimation provides critical information about the distance to objects in the environment, aiding in obstacle avoidance and path planning. Unlike multi-camera or LiDAR-based systems, monocular vision systems are more compact, cost-effective, and widely available in commercial vehicles. Leveraging the advantages of monocular vision alongside pedestrian trajectory prediction reveals new opportunities for scalability, making autonomous technology more accessible and applicable across various platforms.

Integrating object detection and tracking, pedestrian trajectory prediction, and monocular distance estimation holds immense promise for advancing autonomous vehicles’ capabilities by creating a holistic perception system that can better understand the dynamics of the surrounding environment. This integration paves the way for more intelligent decision-making algorithms and ultimately enhances the overall safety, accuracy, and efficiency of autonomous vehicles across a wide range of real-world situations. The pipeline of pedestrian trajectory prediction and distance estimation involves multiple steps:

Detection: A detection model is used to identify and localize the pedestrians in the input video or image stream.

Tracking: Once the pedestrians are detected, a tracking model is used to track their movements over time, linking their positions across multiple frames of the video.

Trajectory Prediction: A trajectory prediction model is applied to predict their future movements based on their current and past positions.

Distance Estimation: In parallel, the data received from the sensors, along with the information provided by the tracking algorithm, are used to infer the distance to the detected pedestrians in each frame.

The multi-stage nature of this unified framework makes the error accumulation within the pipeline a decisive factor in determining the performance of the entire system. The error accumulation effect is commonly observed in frameworks that incorporate multiple deep learning models stacked sequentially. Typically, each individual model within the pipeline expects its inputs to be obtained directly from the environment. If these inputs are produced by the other model, they contain model-produced noise that in turn impairs the performance of the downstream algorithm. This effect is discussed in detail in [5]. In summary, errors in the early stages can propagate to the late stages, leading to inaccurate estimation for downstream applications.

Another challenge arises from the necessity of using multiple large neural networks to perform individual tasks within the pipeline. Apart from the computational complexity, it is essential to ensure that all the models within the pipeline are optimized for their respective upstream inputs, limiting the efficacy of transfer learning techniques.

In this paper, we empirically investigate the influence of errors arising from object detection and tracking on the accuracy of pedestrian trajectory prediction and monocular distance estimation. The present study is divided into two parts: in the first part, we assess the efficacy of the commonly employed object detection and tracking algorithms when applied to a trajectory prediction model. The goal is to evaluate their impact on the performance of predicting the pedestrian trajectories across a range of diverse scenarios.

In the second part, we evaluate two distance estimation algorithms on the custom dataset using the outputs of the upstream object detection algorithm and assess the resulting performance of both models. This experiment is aimed at investigating the impact of error accumulation further. The general framework of our research, along with its quantitative outcomes, are detailed below.

In the field of autonomous driving perception research, previous studies on trajectory prediction have mostly focused on the errors generated by the models in isolation without considering the accumulated errors from object detection and tracking. This omission is primarily due to the use of the existing public datasets, where the ground truth is annotated for single tasks only. To address this issue, we consider the accumulated errors from detection and tracking to investigate the impact of these upstream errors on the downstream trajectory prediction. The main contributions of our work are as follows:

(1) On the pedestrian trajectory prediction task, we demonstrate the impact of detection and tracking errors on the accuracy of prediction using multiple public datasets for training and a custom dataset for evaluation.
(2) On the monocular distance estimation task, we show the effect of error accumulation from detection and tracking using a custom LIDAR-annotated dataset for evaluation and a public dataset for pre-training.

The remainder of this paper consists of five sections. In Section 2, we provide a review of the previous studies related to pedestrian trajectory prediction and distance estimation with a focus on deep learning approaches. In Section 3, we describe the used datasets and evaluation metrics and introduce the detection, tracking, trajectory prediction, and distance estimation approaches we used. In Section 4, we present our results for both experiments and provide an in-depth analysis of our findings in Section 5. Finally, the conclusions are drawn in Section 6.

## 2. Related Work

In this section, we provide an overview of the existing pedestrian trajectory prediction approaches and monocular distance estimation algorithms. Both of these tasks can be solved in numerous ways, creating an extremely vast and diverse research landscape. In our review, we mainly focus on the deep learning approaches due to the progress in this field achieved in recent years.

### 2.1. Pedestrian Trajectory Prediction

There are numerous studies on the topic of pedestrian trajectory prediction for autonomous driving. With the steady development of deep learning technology and the release of public datasets [6,7,8], the research on pedestrian trajectory prediction has been rapidly advancing.

#### 2.1.1. Early Research and Methods

The pedestrian trajectory prediction research started in the 1990s, with the social force model being the most widely used [1]. The early research on pedestrian trajectory prediction was mostly conducted using static scenes and did not fully utilize the dynamic information about the scene. As people’s behavior can be influenced by other pedestrians, an understanding of the intentions of each pedestrian is also required.

Pedestrian detection refers to the ability to identify the presence of a pedestrian in an image or video frame, while tracking focuses on the ability to continuously follow the pedestrian by assigning individual ID (identification) numbers as they move through the scene over time. By combining the information from detection and tracking, it is possible to predict the trajectory of a pedestrian and anticipate their future movements. The combination of trajectory prediction and object tracking technology can improve the prediction accuracy and stabilize the prediction results. The early research on pedestrian trajectory prediction mostly used feature engineering methods combined with machine learning prediction models. The commonly used tracking algorithms include the Kalman filter, kernelized correlation filters, and mean-shift; the commonly used machine learning prediction models include Bayesian inference, hidden Markov models (HMMs), and the Gaussian process. Although these traditional methods have theoretical and mathematical proofs, they are based on specific assumptions. For example, the current state depends solely on the previous state in HMMs and the assumptions made in the Kalman filter, including linearity, normality, and stationarity.

#### 2.1.2. Deep Learning Approaches

The studies on applying deep learning to pedestrian trajectory prediction include a wide range of models with different architectures.

Social GAN: The Social GAN model [9] applied Generative Adversarial Networks (GANs) [10] to the trajectory prediction objective by using a recurrent neural network to observe the previous movement trajectories and predict the future trajectories by training an adversarial discriminator. This was the first model to consider social interactions, thus achieving higher accuracy on the ETH/UCY datasets [6,7] compared to the previously reported results. The main limitations of the model include the use of recurrent networks that are challenging to efficiently parallelize and the training complexity added by the GAN components.

SoPhie: The SoPhie model [11] was a further development of the GAN-based approach that is capable of generating more realistic samples compared to Social GAN. This improvement was achieved by combining all the historical paths in the scene with relevant contextual information, and by employing a bidirectional attention mechanism to learn the most relevant paths. These modifications facilitated further accuracy increases on the ETH/UCY and Stanford Drone datasets [12]. In terms of limitations, SoPhie inherited poor parallelizability and training complexity from Social GAN.

Social-LSTM: The authors in [13] proposed Social-LSTM, which used a recurrent neural network to treat trajectory prediction as a sequence generation task, considering the social interactions of each person in a shared environment.

Traditionally, pedestrian trajectory prediction heavily relied on determining the strength of the interactions between individuals based on their proximity to one another. However, this may not always hold as people further away can still have a higher chance of interacting in the future.

Attention-Based Model: In [14], the authors adopted an attention mechanism to recognize the relative importance of pedestrians at different distances within a moving crowd. Attention-based models can enhance trajectory prediction accuracy by effectively capturing complex social dynamics or the relative importance of each person when navigating in the crowd.

The Graph Neural Network: The graph neural network (GNN) [15,16] is a deep learning method based on graph structures. Since graph structures can effectively represent the relationship and importance between each connected node, they are very suitable for trajectory prediction applications.

Social-BiGAT: It uses the LSTM [17] model to simulate the trajectory of each pedestrian and a graph attention network to simulate the interactions between people and between people and their surroundings [18]. By incorporating graph attention, the model outperformed both Social GAN and SoPhie on ETH/UCY. Along with the limitations introduced by the use of GANs and RNNs, this approach is characterized by increased deployment complexity since many edge-computing devices do not support graph neural networks.

Social-STGCNN: To further improve the representation of social and spatial relations, the authors of [19] proposed a graph convolution-based neural network that interpreted the trajectory data as a spatiotemporal graph. This way, the authors were able to model the social, spatial, and temporal structure of the inputs without using recurrent neural networks, thus alleviating the parallelization limitation of the previously reported approaches. Additionally, instead of using a generative adversarial module for multimodality, Social-STGCNN was designed to output the trajectory distributions directly. Due to these improvements, the authors were able to achieve state-of-the-art (SOTA) performance on the ETH/UCY datasets. However, the use of complex graph convolution-based modules still limits the deployment potential of the model.

#### 2.1.3. Advanced Frameworks

STAR Framework: In [20], the authors proposed a framework called STAR, which models the spatiotemporal characteristics of the data by representing trajectories as graphs. Additionally, STAR utilizes a transformer-based framework [21] to process the time dimension and the space dimension, respectively. The outputs of these two transformer models were joined into a unified spatiotemporal representation. Compared with the traditional recurrent neural networks or convolutional neural networks, STAR demonstrated better performance in high-density crowd scenes with complex interaction relationships, achieving SOTA accuracy on ETH/UCY. On the downside, the STAR framework is also challenging to deploy due to using both graph convolutions and attention-based modules.

Multimodal Trajectory Prediction Framework: In [22,23], the authors proposed a multimodal trajectory prediction framework based on recurrent and convolutional neural networks following the encoder–decoder structure, as well as the multi-view augmentation strategy for generating robust trajectory representations. The multimodality within the model was achieved by producing two-dimensional heatmaps representing the probability distribution of the agents’ locations. Due to the effective topology and augmentation, this framework achieved state-of-the-art performance on the VIRAT/ActEV [24], Stanford Drone [12], and Argoverse [25] datasets. Due to not utilizing graph or attention modules, this model is much more suitable for deployment, and it does not suffer from the mode collapse characteristic of the GAN-based approaches. However, the use of RNNs in the model hinders its parallelization potential, thus increasing the training and inference computational complexity.

Overall, these studies highlight the potential of various deep learning and computer vision techniques to enhance the pedestrian trajectory prediction for autonomous driving. However, most studies are evaluated on public trajectory datasets that provide ideal ground truth tracking data, thereby excluding the impact of detection and tracking errors on the developed trajectory models. In this study, we utilize the SimAug model to assess the effect of tracking and prediction errors on the trajectory prediction accuracy. This model was chosen for its capability to produce multimodal predictions and its overall state-of-the-art performance.

### 2.2. Monocular Distance Estimation

Monocular distance estimation approaches use a single camera to reconstruct the depth information regarding the scene. These methods are less expensive in terms of computational resources and deployment compared to the multi-view distance estimation models, which justifies an increasing research interest in developing these algorithms. Since the task of extracting 3D information from a single 2D frame is a fundamentally ill-posed problem, classical monocular depth estimation algorithms fall behind the multi-view methods in terms of accuracy [26]. Thus, most of the ongoing efforts in the monocular depth estimation research field are concentrated on developing data-driven models that can learn to predict depth from large real-world datasets.

The deep learning landscape for monocular depth estimation consists almost entirely of convolutional neural networks and graph convolutional neural networks. In terms of the training strategies, data-driven depth estimation algorithms can be categorized into supervised, self-supervised, and semi-supervised [26].

#### 2.2.1. Supervised Methods

The models trained in a supervised manner accept images as inputs and output depth or disparity maps. The objective functions of these networks minimize the difference between the generated depth maps and the ground truth depth maps (e.g., maps generated by projecting the LiDAR point clouds onto the image plane). These models are highly accurate but require the data to be completely annotated with pixel-wise distance information, which in turn involves collecting and processing large quantities of LiDAR or radar point clouds, making the training process more expensive in terms of equipment and working hours.

In [27], for example, a Spacing-Increasing Discretization approach was employed to produce fine-grained depth maps. The proposed model uses a dense feature extractor augmented with cross-channel information, as well as multi-scale feature learners. The model also features an ordinal regression optimizer. In essence, the authors reformulated the depth regression problem into a multi-class classification task over distance intervals, thus simplifying the network structure and the training process. As a result, the network does not require additional subsampling and therefore is more computationally efficient. Owing to these improvements over the previously proposed architectures, the model achieved high accuracy on the KITTI [28], Make3D [29], and NYU v2 [30] benchmarks.

In [31], the authors developed the ACAN model: an Attention-Based Context Aggregation Network. This model can derive pixel-wise contextual relations, which increases the quality of the model’s internal representation and therefore results in more accurate depth maps. Their approach relies on a deep residual architecture combined with a dilated layer and a self-attention module that controls the spatial scale of the outputs. Additionally, the self-attention module builds pairwise pixel relationships, thus enhancing the model with rich contextual information. This model achieved competitive performance on the KITTI [28] and NYU v2 [30] datasets.

#### 2.2.2. Self-Supervised Methods

These algorithms obtain depth information automatically, typically by building relations between different input modalities or transformations. The main benefit of the self-supervised models comes from their independence on the annotated data. This property facilitates the use of larger datasets that are easier to collect but also results in several complications. In particular, these models often require a prominent level of diversity in the data to achieve a sufficient level of generalization.

As for the specific approaches, the Monodepth and Monodepth2 [32,33] models are well-known examples of a self-supervised depth estimation framework. These models are built using a residual feature extraction model combined with a pose estimation network. The latter is used to infer pose deviations between the stereo image pairs. Based on the visual and pose features, a disparity map is produced in a self-supervised manner and then optionally rescaled to meters using the real-world geometric configuration of the cameras. Since the model relies on stereo image pairs and the camera parameters to convert disparity to depth, its application to real-world scenarios with different settings is limited. Both models achieved SOTA results on the KITTI benchmark.

Another self-supervised model by Guizilini et al. [34] relies on three-dimensional convolutions that extract depth information via processing the spatial image features. The model uses symmetrical packing and unpacking blocks: novel deep learning modules that are designed to combine the information contained within the encoder and decoder features. Unlike Monodepth2, PackNet relies solely on monocular sequences of frames and externally defined depth scaling factors; this property makes the model easier to apply to a custom dataset, resulting in improved performance on the KITTI dataset.

#### 2.2.3. Semi-Supervised Methods

Semi-supervised models can be viewed as a combination of the previous two: they require large unlabeled datasets for feature extraction training and small labeled datasets for matching the outputs to the distance estimation objective. An example of such an approach can be found in [35], where the authors developed a semi-supervised method of estimating depth maps that relies on geometry-aware symmetric domain adaptation (GASDA). The model addresses the generalization problem by using synthetic training data. The method uses image translating combined with monocular depth prediction via the use of generative neural networks, namely the CycleGAN model [36]. The network is trained by utilizing two separate image style translations and a set of symmetric depth estimation sub-networks. GASDA leverages the stereo epipolar geometry, resulting in accurate 192 × 640 depth maps. This model demonstrated strong performance on both the KITTI and Make3D datasets.

In this study, we additionally separate a subset of the depth estimation algorithms that predict the object-specific distance instead of generating depth maps. These models are typically supervised and provide the user with single distance values for each object detected in the image frame. In our previous works, we proposed a lightweight convolutional framework capable of predicting the object-specific distances from monocular images: the CDR model [37,38]. This model was designed as a simple and efficient solution that is easier to train and deploy compared to its depth estimation counterparts and was shown to achieve an accuracy comparable to the Monodepth2 model on the KITTI dataset.

In our experiments, we incorporate the CDR and the Monodepth2 models within a unified detection–tracking–prediction framework. This choice was made due to the substantial structural differences between these approaches. By comparing both models, we facilitate a more general assessment of the impact of detection and tracking errors on distance prediction accuracy.

### 2.3. Errors in Trajectory Prediction and Distance Estimation

The quantification of the errors in pedestrian trajectory prediction and monocular distance estimation tasks is a complex problem that can be solved in several ways.

For trajectory prediction, the most typically employed performance metrics include the average displacement error (ADE) and final displacement error (FDE). The ADE measure is defined as the average $L_{2}$ (denoted as $\left|\right|\cdot {\left|\right|}_{2}$) distance between the predicted trajectory points and the ground truth. In the case of multimodal trajectory prediction, two types of this measure can be used: ${\mathrm{ADE}}_{K}$ and minADE. The former is computed as the average ADE for *K* sampled trajectories for a given agent, and the latter stands for the minimum ADE among the *K* samples [23,39]. The FDE metric, in turn, measures the $L_{2}$ distance between the endpoint of the predicted trajectory and the ground truth. Similarly to ADE, the FDE measure can be computed as ${\mathrm{FDE}}_{K}$ or FDE [39].

Apart from these measures, various more complex approaches may be used to quantify prediction errors, such as time-to-collision (TTC) or the driving reliability and error analysis method (DREAM) [40]. The authors of DREAM proposed a comprehensive framework for identifying crash causation patterns based on the phenotype–genotype scheme. The phenotype in the framework is the direct contributing factor of the crash, while the genotype is the latent contributing factor. While being much more informative and interpretable, this method is more suitable for crash case studies and is challenging to apply in our experimental setting.

In our study, we adopt minADE and minFDE as performance metrics for trajectory prediction due to the widespread use of these measures, which facilitates easier comparison to the existing approaches.

The formulae for minADE and minFDE are provided in Equations (1) and (2), where *N* stands for the batch size, *T*, and *h* denote the overall temporal trajectory length and the history length, respectively, and *K* is the number of sampled predicted trajectories. In both equations, *Q* denotes the ground truth trajectory points (*x* and *y*), and $\hat{Q}$ stands for the predicted trajectory points.

(1) $$\mathrm{minADE}=\frac{1}{N(T-h)}\sum_{i=1}^{N}min_{K}\sum_{t=h+1}^{T}\left|\right|Q_{t}^{i}-{\hat{Q}}_{t}^{i}{\left|\right|}_{2}$$

(2) $$\mathrm{minFDE}=\frac{1}{N}\sum_{i=1}^{N}min_{K}\left|\right|Q_{T}^{i}-{\hat{Q}}_{T}^{i}{\left|\right|}_{2}$$

In both equations, the minimum operation is used over the set of predicted sample trajectories to estimate the model’s reconstruction performance. This step is performed to exclude the samples that realize the alternative modes of the agents’ behavior.

For monocular distance estimation, the choice regarding the error measures is just as diverse. The most commonly used metrics include mean absolute error (MAE) or absolute relative distance (ARD), root mean squared error (RMSE), and logarithmic RMSE [26]. All these measures compute the mean error in meters or fractional units relative to the ground truth distance and mostly differ in the handling of large outliers. The RMSE metric, for instance, tends to amplify the role of large-valued errors [38]. Additionally, in [41], the range error and the relative range error measures were proposed. These metrics are derived for the stereo vision applications and take the stereo parameters of the vision system into account, resulting in more accurate error estimation.

For our experiments, we adopt mean absolute error (MAE), as well as the standard deviation of the error (STD) and its 95th percentile. MAE is calculated according to Equation (3), where *z* is the ground truth distance and $\hat{z}$ is the predicted distance.

(3) $$\mathrm{MAE}=\frac{1}{N}\sum_{i=1}^{N}\left|z^{i}-{\hat{z}}^{i}\right|$$

This combination of metrics was chosen for the following reasons:

– The mean absolute error is the standard evaluation metric for the task. It facilitates the quantitative and qualitative comparison with other reported results on monocular distance estimation.
– The standard deviation of mean absolute error quantifies the consistency of the model’s outputs and thus characterizes the reliability of the chosen method on a given dataset.
– The 95th percentile of MAE provides deeper insight into the structure of the model’s error distribution and can be viewed as the upper error bound, i.e., the worst-case error computed excluding the outliers.

## 3. Materials and Methods

The task of trajectory prediction can be considered as a downstream task of detection and tracking. Therefore, errors accumulated from detection and/or tracking will be passed on to the trajectory prediction models, leading to inaccurate outcomes. The same effect, which we refer to as error accumulation, is generally true for the distance estimation task. In this section, we describe the datasets that were used in our experiments, the chosen evaluation metrics, and the pipelines that we evaluated.

### 3.1. Datasets

#### 3.1.1. Trajectory Prediction

In the trajectory prediction experiment, we used three datasets for training and validation: Argoverse 1 [25], BDD100K [42], and KITTI [28].

The Argoverse 1 dataset was released in June 2019 and contains road information from Miami and Pittsburgh. The videos in the dataset have a resolution of 1920 × 1200 and are recorded at a rate of 30 Hz, with lengths ranging from 15 to 30 s. We preprocessed the tracking data into trajectory data, resulting in 1372 training samples and 406 validation samples when applying a 2.5 FPS frame rate.

The BDD100K dataset was released in May 2018 and contains road information collected from various locations in the US, including New York, Bay Area, Berkeley, and San Francisco. The videos in the dataset have a resolution of 1280 × 720 and are recorded at a rate of 30 Hz, with each video lasting 40 s. After applying a 2.5 FPS frame rate sampling, the data contain 685 training and 205 validation samples.

The KITTI dataset was released in 2012 and contains road information from Karlsruhe, Germany. The videos in the dataset have a resolution of 1242 × 375 and were recorded at a rate of 30 Hz. We used video data from the subsets 0015, 0017, and 0019 as the training set, while video data from 0016 were used as the validation set. There are 686 training and 213 validation samples in the dataset after a 2.5 Hz frame rate sampling is applied.

**Figure 1:**
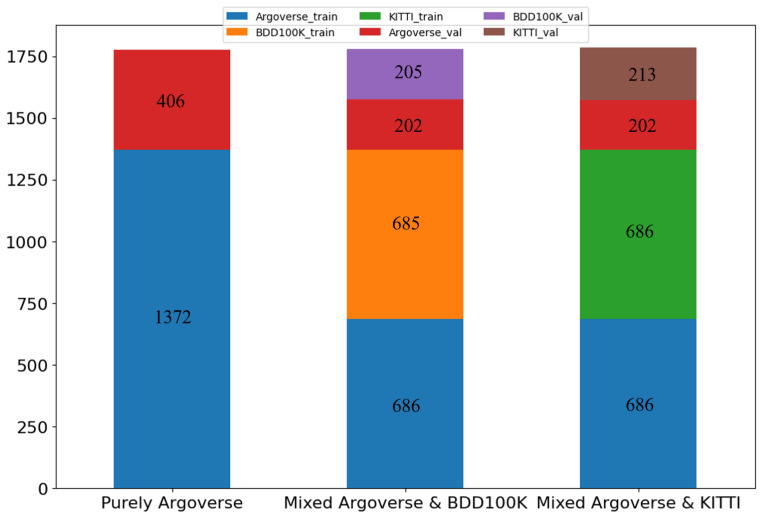
The quantity and the ratio of the training set and validation set.

For model training, we used three different datasets with varying ratios. The first set consisted of 1372 training and 406 validation samples from the Argoverse 1 dataset, the second set had 1371 training and 407 validation samples from Argoverse 1 and BDD100K datasets, and the third set had 1372 training and 415 validation samples from Argoverse 1 and KITTI datasets. Figure 1 shows the quantities and ratios. We evaluated the trained models on various self-gathered Taiwan traffic scenes, including the Xindian district in New Taipei City, two scenes in Yonghe District (#1 and #2), New Taipei City, and Daan District in Taipei City.

In this study, the prediction of the next 12 frames of pedestrian trajectories was based on observing 8 history frames of trajectories, with a 2.5 FPS frame rate, resulting in the observation of 3.2 s to predict the following 4.8 s.

#### 3.1.2. Distance Estimation

For the distance estimation experiment, the models were pretrained on the Eigen split [43] of the KITTI dataset and evaluated on a custom dataset collected in Taichung City and annotated with the LIDAR ground truth. This dataset consists of a single video with a total duration of approximately 9 min, a frame rate of 30 FPS, and a 1280 × 720 resolution. Out of all the video frames, 7064 have corresponding point clouds, roughly 19,400 points in each. To match the CDR ground truth format as described in [37], the following procedures were performed:

(1) In each video frame, road agents belonging to the classes “person”, “car”, “bicycle”, “motorcycle”, “bus”, “train”, and “truck” were detected using the YOLOv7 object detector.
(2) For each frame, if the corresponding LIDAR point cloud was recorded, the points were projected onto the image plane using the pinhole camera projection model.
(3) For each bounding box, if the number of distance points within its boundaries was larger than 50, the bounding box was denoted as having the ground truth distance. Otherwise, the ground truth distance for this bounding box was set to be non-existent (nan).
(4) For the bounding boxes with the ground truth distance, the distance points were arranged in ascending order, after which the smallest 15% (distance-wise) of the points were averaged. This average was determined to be the ground truth distance for a given object.

Following these procedures, a dataset consisting of 23,478 objects with corresponding distances was created. It can be noticed that the ground truth object distances were obtained using a slightly different approach compared to the KITTI data. This algorithm was designed after extensive empirical testing as the one producing the most visually plausible ground truth values.

The obtained samples were split into training, validation, and testing sets frame-wise. The split was performed preserving the chronological frame order; i.e., all the objects in the validation set appear later in the video than those in the training set. This decision was made to minimize the similarity between training and testing data and to ensure a fair comparison. The dataset statistics are shown in Table 1.

### 3.2. Pedestrian Trajectory Prediction Pipeline

**Figure 2:**
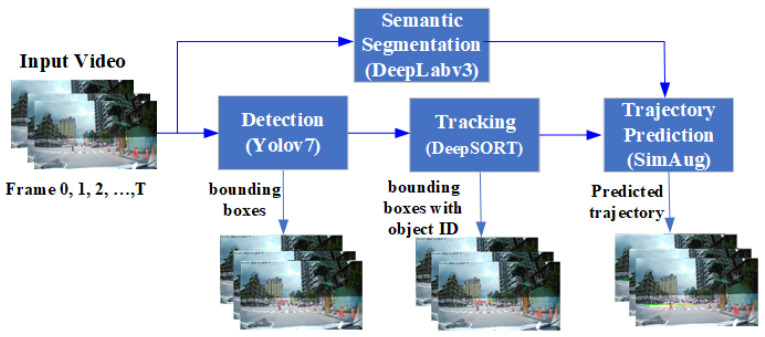
The workflow of the trajectory prediction experimental system.

We adopted popular object detection and tracking models, namely YOLOv7 [44] and DeepSORT [45], to calculate observed trajectories from videos instead of using ground truth from the datasets. Pedestrian information includes a pedestrian ID (tracking number) and the center point of the detected bounding box in each image. In addition, we utilized a pretrained trajectory prediction model and data augmentation from [22,23] and performed fine-tuning using custom datasets. The system workflow is illustrated in Figure 2.

In Figure 2, the scene semantic segmentation is used for extracting high-level features from videos to provide a consistent and high-level scene understanding, enhancing the model’s ability to predict trajectories across varying conditions. At test time, segmentation features are extracted from real videos using a pretrained DeepLabv3 model. These features are then used to predict future trajectories in real-world unseen videos.

During training, we set the epoch and batch size to 200 and 20, respectively. To optimize the model, we chose the Adadelta optimizer [46]. The optimizer’s initial learning rate was set to 0.6, and it decayed by 0.05 every 2 epochs. We evaluated the model’s performance and stored its parameters every 500 steps by feeding them into the validation dataset.

### 3.3. Monocular Distance Estimation Pipeline

For this task, consistent with our previous works, we use the YOLOv7 object detection model to obtain the bounding box predictions. For distance estimation, we use the Convolutional Distance Regression (CDR) model [38] and the Monodepth2 model [33] to study the impact of error accumulation and the efficacy of transfer learning on both distance and depth estimation algorithms.

The CDR model is pretrained on the KITTI dataset using the optimal structural and training settings determined in [38] (random downsampling, weighted Kullback–Leibler Divergence Loss, crop factor of 0.3, β=0.99, base decoder) and fine-tuned on the training set for 50 epochs with a learning rate of 0.00007 and a batch size of 640 using Adam optimizer [47]. A stepwise learning rate scheduler is used to control the loss dynamics: the learning rate is decreased by a factor of 2 every 10 epochs. Unweighted Kullback–Leibler Divergence is used as a loss function. We refer to this procedure as pipeline-aware fine-tuning since the model is pretrained using the outputs of the upstream detection model.

The Monodepth2 model used in this experiment is pretrained on the KITTI dataset in the stereo + mono mode without any additional fine-tuning. The model was not fine-tuned due to the monocular nature of the dataset: in our experiment, only the monocular training is available for the model, and it does not support metric depth map generation.

Since certain classes of objects (namely “train”, “bus”, “truck”, and “bicycle”) are either not present in the dataset or heavily under-represented, they are excluded from the evaluation on the test set, leaving only the “person”, “bike”, and “car” classes.

## 4. Results

### 4.1. Experiment 1: Pedestrian Trajectory Prediction

The performance of the trajectory prediction pipeline on various datasets is shown in Table 2, where the error values are provided in pixels. The resolution of the test set is 1920 × 1080 and recorded at a rate of 30 Hz. We resized the input image to 608 × 608 pixels in applying the Yolov7 detection model. Our data preprocessing and standardization procedure includes several key steps. Initially, we analyze the original dataset’s annotation information to identify videos containing pedestrians and downsample these to 2.5 FPS. We then exclude videos with fewer than twenty frames following the downsampling as our study requires eight frames for observation and twelve for prediction. For the retained videos, we adjust the bounding box sizes to match the downsampled index values and store the pedestrian index values, tracking information, and bounding box center points as trajectory data. Using the downsampled index information, we generate corresponding images, resize them to a 1920 × 1080 resolution, and apply semantic segmentation using a pretrained model combining an Xception backbone network with a Deeplabv3 semantic segmentation network. Finally, we package the semantic segmentation data and pedestrian trajectory data into a model-suitable format, structuring the dataset to use eight frames for observation and the subsequent twelve frames for prediction. This standardized approach ensures consistency in our data preparation, facilitating robust trajectory prediction and analysis.

The results are presented using two metrics: minADE and minFDE. The values of these metrics are provided in pixels since both the training and testing datasets used for evaluation do not provide depth-wise annotation (i.e., LIDAR point clouds), thus making the transformation of the image points into real-world coordinates impossible. Although this circumstance limits the qualitative interpretation of the results, it is still possible to estimate the impact of the tracking and detection errors on the accuracy by comparing the obtained results to the results reported on the benchmark datasets that were used in this study. We provide further details regarding this comparison in Section 5.

If we only consider the trajectory prediction without accounting for the impact of detection and tracking like in previous studies, the results are 67.9 (pixels) for minADE and 175.6 (pixels) for minFDE, which is more optimistic than the practical scenario. When the detection and tracking errors are included, minADE and minFDE increase to 133.8 (pixels) and 285.8 (pixels), respectively. The accuracy of trajectory prediction is closely related to many safety features of autonomous driving or advanced driver assistance systems. Overly optimistic error analysis may affect the downstream safe path planning.

**Figure 3:**
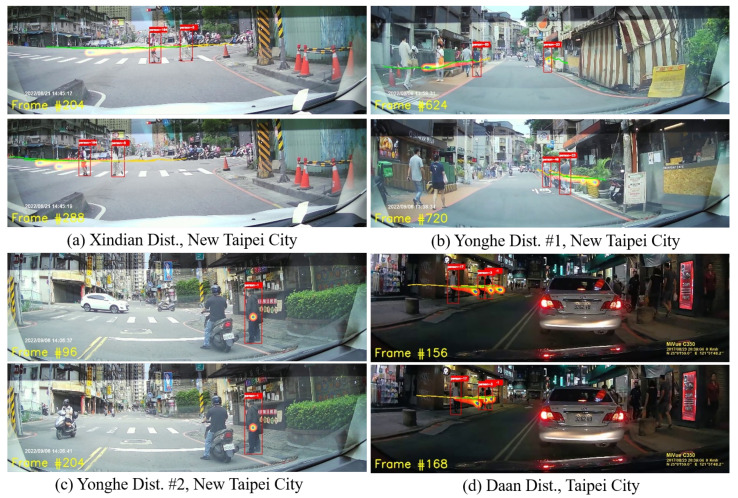
Trajectory prediction pipeline: visualization.

The visualization of the pipeline’s outputs appears in Figure 3. For inference on these samples, the model was trained using the complete Argoverse 1 dataset. The red bounding boxes provide information about the pedestrians’ location, the yellow lines represent the model’s observation of the pedestrians in the past 3.2 s, the green lines display the ground truth trajectories of the pedestrians in the next 4.8 s, and the heatmaps show the predicted trajectories in the next 4.8 s. Additionally, the text in the bottom left indicates the frame index.

### 4.2. Experiment 2: Monocular Distance Estimation

The performance of the CDR- and Monodepth2-based distance estimation pipelines pretrained on the KITTI dataset and evaluated on the custom dataset are presented in Table 3 and Table 4. In the tables, the results are provided in the form “MAE (m)/STD of MAE (m)/95th percentile of MAE (m)”. For instance, the table entry “5.27/6.49/14.81” can be interpreted as “the model has the mean absolute error of 5.27 (m) with the standard deviation of 6.49 (m), and 95% of all errors are less than or equal to 14.81 (m)”.

**Figure 4:**
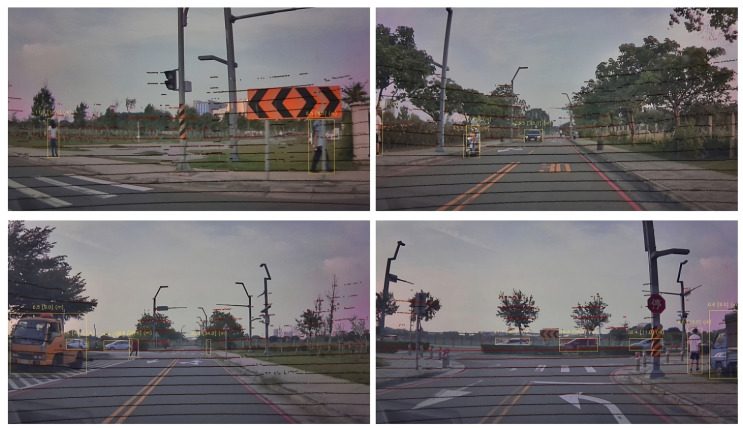
Visualization of monocular distance pipeline.

The examples displaying the CDR model’s performance are shown in Figure 4, where the red dots represent the LIDAR points, the distances in square brackets represent the ground truth, and the distances without brackets represent the model’s predictions.

## 5. Discussion

The minADE/minFDE results obtained in Experiment 1 (Table 2) show that the performance of the state-of-the-art Multiverse+SimAug model is noticeably hindered when applied to an unfamiliar dataset and using the outputs of the upstream tracking model instead of the ground truth bounding boxes. Meanwhile, on the benchmark datasets, the model achieves minADE values on the order of 20 (px) and minFDE values of 40 (px) [23]; regarding our custom data and within the tracking pipeline, these values are 5–7 times higher for almost all the cases. This indicates that the impact of the error accumulation on the trajectory prediction accuracy cannot be omitted in real-world applications. Moreover, this effect is clearly visible in Figure 3: the heatmaps of the predicted trajectories, while generally following the ground truth tracking curves, are noticeably different from the latter.

The same impact of the error accumulation can be observed in Experiment 2 (Table 3 and Table 4). This is especially clear when studying the standard deviations of the errors and the 95th percentiles: both quantities for both models are equal to or larger than the average errors. For comparison, on the KITTI dataset, both Monodepth2 and CDR yield errors on the scale of 2 (m) [38], while, on an unfamiliar dataset, the MAE values are several times higher (4.62 (m) for the CDR model and 4.80 (m) for Monodepth2).

This experiment also demonstrates the impact of pipeline-aware fine-tuning on the performance of the distance estimation algorithm. The fine-tuned CDR model yields lower error values compared to Monodepth2 for the “car” and “motorcycle” classes, as well as for the middle-distance range from 20 to 40 (m), despite being much simpler in its structure. Additionally, the 95th percentile of MAE is lower for the CDR model when viewed within the class-wise distribution. It is reasonable to assume that the overall better performance of the CDR model is due to fine-tuning. The error rates for Monodepth2 may be reduced by fine-tuning as well; however, this would require much more data with binocular modality, which may be substantially more expensive in terms of the deployment costs and computational complexity.

## 6. Conclusions

This study demonstrated the importance of accurate detection and tracking in the development of effective pedestrian trajectory prediction and monocular distance estimation models for autonomous driving. For the trajectory prediction task, we employed a combination of multiple models, including the detection model YOLOv7, the tracking model DeepSORT, the trajectory prediction model Multiverse, and the data augmentation method SimAug. We demonstrated that detection and tracking errors can have a substantial impact on the accuracy of trajectory prediction. For the monocular distance estimation task, we combined the YOLOv7 object detection model with Monodepth2 and the CDR monocular distance estimation models and demonstrated that the accuracy of the detection, as well as the similarity of the dataset to the training data, have a noticeable effect on the MAE values, as well as on the consistency of the predictions (standard deviation) and the width of the error distribution.

Overall, our findings suggest that improving the detection and tracking capabilities should be a key priority for developers of autonomous driving systems as these factors are critical to ensuring the safety and reliability of these systems in real-world environments. Additionally, deeper integration between models is required to enhance the positive effect of fine-tuning, ideally by creating a single multi-modal framework that jointly performs detection, tracking, trajectory prediction, and distance estimation.

## Figures and Tables

**Table 1:** Number of frames and objects in the custom distance estimation dataset.

Subset	Frames	Objects
training	4500	14,760
validation	1025	3487
testing	1539	5231

**Table 2:** The performance of the trajectory prediction pipeline trained on the tracking model’s outputs.

Train/Val Set	Test Set	minADE (px)	minFDE (px)
Argoverse1	Xindian Dist.	133.8	285.8
	Yonghe Dist. #1	80.8	215.7
	Yonghe Dist. #2	8.5	16.2
	Daan Dist.	55.9	116.5
Argoverse1 and BDD100K	Xindian Dist.	96.3	181.5
	Yonghe Dist. #1	98.9	255.1
	Yonghe Dist. #2	12.9	7.4
	Daan Dist.	69.8	158.2
Argoverse1 and KITTI	Xindian Dist.	108.9	251.2
	Yonghe Dist. #1	113.0	295.4
	Yonghe Dist. #2	23.5	77.4
	Daan Dist.	71.9	137.0

**Table 3:** The distance estimation pipeline performance: class-wise distribution.

Class	Samples	CDR Model	Monodepth2
person	1436	5.27/6.49/14.81 ^1^	5.11/6.77/16.88
car	2535	4.04/4.84/13.25	4.40/5.55/15.12
motorcycle	452	5.77/7.53/16.04	6.06/6.52/20.72
overall	4423	4.62/5.78/14.16	4.80/6.10/16.46

^1^ The results are provided in the form MAE (m)/STD of MAE (m)/95th percentile of MAE (m).

**Table 4:** The distance estimation pipeline performance: distance-wise distribution.

Distance (m)	Samples	CDR Model	Monodepth2
$\left[0,10\right)$	1182	2.54/3.95/9.27 ^1^	1.65/3.58/3.79
$\left[10,20\right)$	1544	3.54/3.78/9.50	3.46/4.98/10.67
$\left[20,40\right)$	1600	5.87/4.54/14.16	7.87/6.40/19.27
$\left[40,+\infty \right)$	97	26.45/13.79/48.95	13.88/9.08/27.09
overall	4423	4.62/5.78/14.16	4.80/6.10/16.46

^1^ The results are provided in the form MAE (m)/STD of MAE (m)/95th percentile of MAE (m).

## Data Availability

The Argoverse dataset can be currently downloaded from https://www.argoverse.org (accessed on 1 June 2023). The BDD100K dataset is available at https://doc.bdd100k.com/download.html (accessed on 1 June 2023), and the KITTI dataset can be accessed via https://www.cvlibs.net/datasets/kitti/ (accessed on 1 June 2023). The listed datasets are publicly available and were published under the Creative Commons Attribution-NonCommercial-ShareAlike 3.0 License.

## Abbreviations

The following abbreviations are used in this manuscript:

MDPI	Multidisciplinary Digital Publishing Institute
DOAJ	Directory of open access journals
ADAS	Advanced Driver Assistance System
KCF	Kernelized Correlation Filter
ADE	Average Displacement Error
FDE	Final Displacement Error
minADE	Minimum Average Displacement Error
minFDE	Minimum Final Displacement Error
MAE	Mean Absolute Error
STD	Standard Deviation
CNN	Convolutional Neural network
LSTM	Long Short-Term Memory
GNN	Graph Neural Network
GAN	Generative Adversarial Network
